# Ancora: a web resource for exploring highly conserved noncoding elements and their association with developmental regulatory genes

**DOI:** 10.1186/gb-2008-9-2-r34

**Published:** 2008-02-15

**Authors:** Pär G Engström, David Fredman, Boris Lenhard

**Affiliations:** 1 Computational Biology Unit, Bergen Center for Computational Science, University of Bergen, Thormøhlensgate, N-5008 Bergen, Norway; 2 Sars Centre for Marine Molecular Biology, University of Bergen, N-5008 Bergen, Norway; 3 Programme for Genomics and Bioinformatics, Department of Cell and Molecular Biology, Karolinska Institutet, S-17177 Stockholm, Sweden

## Abstract

Metazoan genomes contain arrays of highly conserved noncoding elements (HCNEs) that span developmental regulatory genes and define regulatory domains. We describe Ancora , a web resource that provides data and tools for exploring genomic organization of HCNEs for multiple genomes. Ancora includes a genome browser that shows HCNE locations and features novel HCNE density plots as a powerful tool to discover developmental regulatory genes and distinguish their regulatory elements and domains.

## Abstract 2

Ancora is a web resource that provides data and tools for exploring genomic organization of highly conserved noncoding elements for multiple genomes.

## Rationale

Comparisons of metazoan genome sequences have revealed an abundance of genomic segments that are highly conserved across large evolutionary distances even though they do not encode proteins and do not tend to be near transcription start sites. For example, 256 non-exonic segments longer than 200 bp were found to be perfectly conserved between human, mouse and rat genomes; 140 of these were more than 10 kb away from any known gene [1]. Using less stringent criteria for length and sequence similarity, other investigators have found thousands of non-exonic segments in the human genome that are conserved in organisms as distant as fugu [2,3] and shark [4].

Several lines of evidence indicate that these highly conserved noncoding elements (HCNEs) play a fundamental role in regulating animal development and constraining genome evolution. In vertebrates, insects and worms, HCNEs tend to cluster in the vicinity of developmental regulatory genes [1-7]. Through experiments in transgenic animals in which cloned HCNEs are tested for the ability to drive transcription of a reporter gene, many HCNE sequences have shown the ability to induce part of the embryonic expression pattern of a developmental regulatory gene located in the genomic neighborhood of the endogenous HCNE [3,8-11]. These experiments have associated HCNEs and developmental genes separated by considerable genomic distances, up to 800 kb in human [8], suggesting that many HCNEs act as long-range regulatory elements. Hundreds of HCNEs have now been characterized as developmental enhancers in transgenic mice, frogs or zebrafish, and the list is growing rapidly [10,12-14].

The emerging model for explaining these observations is that an array of HCNEs defines a region of regulatory inputs of its target gene(s), and that the full complement of those inputs results in the expression pattern of the gene [3,8-11]. If this notion that HCNE arrays constitute regulatory domains is correct, chromosomal rearrangements within HCNE arrays should be selected against in evolution [15-17]. Accordingly, large HCNE arrays have been found to correspond to the largest and most deeply conserved blocks of synteny across vertebrates [18] and across insects [6]. In addition to HCNE arrays and their target genes, many of these synteny blocks contain unrelated (bystander) genes that do not appear to be regulated by the HCNEs, although they can be situated between HCNEs and target genes, as well as contain HCNEs in their introns. Kikuta *et al*. [18] termed these synteny blocks 'genomic regulatory blocks' (GRBs) and demonstrated that, for some GRBs, it is possible to distinguish bystander from target genes by comparing mammalian genome sequences with those of teleost fish (such as fugu and zebrafish). This is facilitated by a whole-genome duplication event that occurred in the teleost lineage [19] and caused each GRB to be present in two copies, thereby allowing some bystander genes to be disentangled from HCNE arrays during the subsequent rediploidization [18].

Despite a rising interest in HCNEs in the genomics and evo-devo community, there has been a lack of resources that provide information about HCNEs and allow researchers to explore the distribution of HCNEs along chromosomes. Here, we describe Ancora [20], a web resource consisting of: a genome browser where HCNE locations and HCNE density plots can be viewed over different genomes, with a number of adjustable parameters; data files that allow users to easily view HCNE locations and densities in the UCSC Genome Browser [21]; and a service that allows users to view HCNE data in the Ensembl browser [22] through the distributed annotation system (DAS) protocol for sharing sequence annotations [23]. We demonstrate how Ancora can be used to discover developmental regulatory genes and distinguish their chromosomal regulatory domains that correspond to the GRBs described above. The visualization of these regulatory domains is the most powerful and novel function of Ancora. We anticipate that Ancora will be particularly useful for assigning distal regulatory elements to their target genes, and for the discovery of hitherto unknown developmental regulatory genes, including noncoding RNAs.

## A comprehensive HCNE database

**Figure 1:**
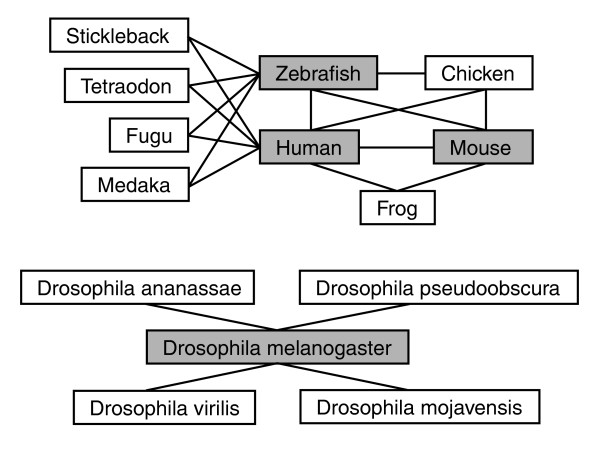
Comparisons available in Ancora. Shaded boxes correspond to genomes shown in the Ancora genome browser. Connecting lines indicate pairwise genome comparisons for which HCNEs are available in Ancora. The following genome assemblies underlie the current data sets: human NCBI 36, mouse NCBI 36 and 37, chicken v2.1 [41], *Xenopus tropicalis *v4.1 (US DoE Joint Genome Institute), zebrafish Zv6 and Zv7 (The Wellcome Trust Sanger Institute), fugu v4.0 [42], *Tetraodon nigroviridis *V7 [19], stickleback v1.0 (The Broad Institute), medaka v1.0 [43], *D. melanogaster *rel. 5 [44], *D. pseudoobscura *rel. 2 [45] and the February 2006 releases of *D. ananassae*, *D. virilis and D. mojavensis *[46].

Ancora rests on a database of HCNEs conserved between various metazoan genomes (Figure 1). Building on our previously described strategies for detecting HCNEs [2,6,18] we have created a refined procedure that is not biased against a chosen base genome and better captures HCNEs duplicated in genome evolution. We identify HCNEs by scanning pairwise BLASTZ net whole-genome alignments (nets) [24] downloaded from the UCSC Genome Browser database [21] for regions with at least *I *identities over *C *alignment columns. Because different similarity criteria may be appropriate for different loci and investigations, we scan for conserved elements using at least two different window sizes (*C *= 30 and *C *= 50) and several different similarity thresholds (*I*/*C*) in the range 70-100% for each species pair. The algorithm that creates net alignments is designed to retain only the best alignment for each position in one of the genomes [24]. For each pairwise comparison, we therefore scan two sets of nets (one from the perspective of each genome) in order not to miss elements duplicated in either lineage. This is particularly important for comparisons between teleost fish and other vertebrates, because of the whole-genome duplication that occurred in the teleost lineage [19]. We subsequently merge highly conserved elements that overlap on both genomes, but not elements that coincide on only one of the genomes, so that duplicated elements remain distinct. After discarding elements whose genome coordinates overlap by one or more base-pairs with annotated exons, we remove repetitive sequences by considering overlap with known repeats and the number of high-identity alignments obtained by realignment of each sequence against the two respective genomes. We consider remaining elements as HCNEs. The exon and repeat annotations we use, and the realignment parameters we employ, are listed on the Ancora web site, where an up-to-date description of our HCNE detection procedure is maintained. To illustrate the effect of parameter changes on the number of HCNEs detected, Table 1 lists HCNE counts for some selected settings and genomes.

**Table 1:** Counts for selected HCNE sets

Criteria for HCNE detection		Number of HCNEs detected in indicated comparison			
	
Minimum identity	Minimum size (bp)	Human vs mouse	Human vs chicken	Human vs zebrafish	Zebrafish vs *Tetraodon*
80% over 30 columns	30	NC	125,174	19,596	57,681
90% over 30 columns	30	NC	78,831	8,260	26,157
96% over 30 columns	30	305,015	50,478	3,656	10,205
100%	30	150,487	35,338	1,721	4,737
70% over 50 columns	50	NC	93,162	16,725	45,828
80% over 50 columns	50	NC	63,304	7,169	25,997
90% over 50 columns	50	265,537	36,794	3,127	8,610
95% over 50 columns	50	107,860	22,530	1,228	3,078
98% over 50 columns	50	68,600	17,579	763	1,782
100%	50	34,785	11,934	330	754
90% over 50 columns	100	81,065	15,339	733	1,695
95% over 50 columns	100	25,801	7,901	188	450
100%	100	4,919	2,475	20	61
100%	200	494	365	0	2

Counts indicate the number of HCNEs obtained by collapsing HCNEs onto the assembled chromosomes of selected reference genomes. HCNEs were counted in this way to reduce redundancy and thereby make counts more comparable between data sets. The underlying Ancora data sets are not biased by selecting either genome as a reference. Note that HCNEs are generally larger than the window size (30 or 50) used to identify them because the procedure that detects HCNEs merges overlapping conserved elements. NC, not calculated.

## Exploring HCNEs and GRBs with the Ancora genome browser

**Figure 2:**
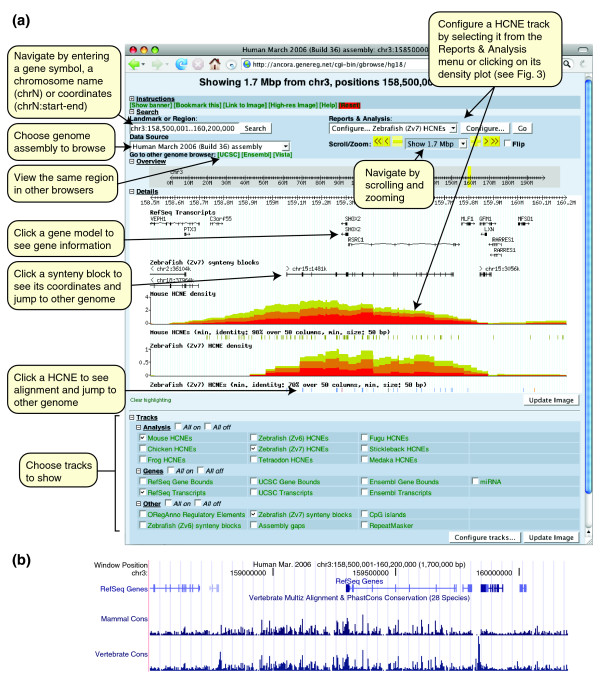
A 1.7 Mb region around the human *SHOX2 *gene. **(a) **Ancora genome browser main view. *SHOX2*, a homeobox gene implicated in limb development [47], is embedded in an array of HCNEs detected by comparison with mouse and zebrafish genomes. Overlaid density plots show densities of HCNEs detected at similarity thresholds of 95% (yellow), 98% (orange) and 100% (red) in the mouse comparison and similarity thresholds of 70%, 80% and 90% in the zebrafish comparison, over a 50 column sliding window. Note that the density of the most strongly conserved HCNEs (red) peaks around *SHOX2*. Synteny blocks are based on net alignments with the zebrafish genome [18]; boxes indicate aligned segments, connecting lines indicate gaps and labels indicate alignment orientation and position in the zebrafish genome assembly. The centrally shown synteny block encompasses *SHOX2*, *RSRC1 *(a gene of unknown function) and the array of HCNEs conserved in zebrafish. **(b) **Conservation profiles for the same region in the UCSC Genome Browser [21]. Comparison between (a) and (b) demonstrates qualitatively different information provided by the HCNE density plots in (a).

Ancora contains a genome browser designed to explore the distribution of HCNEs on metazoan chromosomes (Figure 2a). The browser is currently set up to show the genomes of human, mouse, zebrafish and *Drosophila melanogaster*; we aim to expand this list in the future.

### Basic usage

To put HCNEs in context, the browser also shows gene annotation from NCBI [25], Ensembl [22], the UCSC Genome Browser [21], Mouse Genome Informatics [26], the Zebrafish Information Network [27], FlyBase [28] and miRBase [29], as well as a selection of other annotation tracks from UCSC. The user can click on gene models to bring up detailed gene information pages from the original data sources. By default, the HCNEs are colored by the chromosome they align to in the other genome. This simplifies the identification of conserved HCNE arrays: a stretch of HCNEs in the same color suggests a conserved array. To visualize the tendency of HCNE arrays to correspond to large synteny blocks, we have included tracks showing human-zebrafish synteny blocks and *Drosophila *synteny blocks from recent analyses [6,18]. (The human-zebrafish synteny blocks should be interpreted with caution, however, because of artifacts in the underlying zebrafish genome assembly - in particular artificial segmental duplications, which may appear as overlapping synteny blocks on the human genome.) The user can move between the vertebrate genomes that the genome browser displays by clicking on HCNEs and synteny blocks, which link aligned regions from the different genomes. Ancora also provides links that bring up the same region in other major genome browsers (Ensembl, UCSC and FlyBase) and the VISTA browser, which is useful for detailed examination of sequence conservation [30].

### GBrowse extensions in Ancora

**Figure 3:**
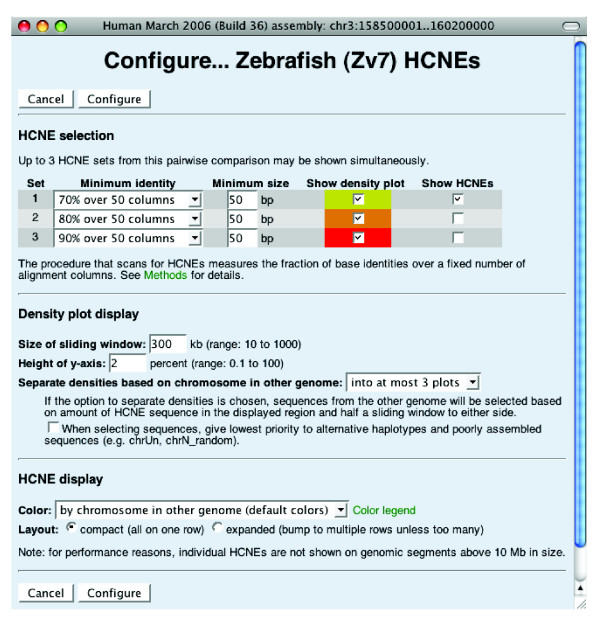
HCNE track configuration page. Up to three HCNE sets from each pairwise comparison can be shown simultaneously. A set is selected by choosing a similarity threshold (for example, 70% identity over 50 alignment columns), and can be further restricted by an arbitrary threshold on HCNE size. Note that HCNEs may be larger than the window size (30 or 50 columns) used to identify them because the procedure that detects HCNEs merges overlapping conserved elements. For each selected set, the user can choose to see HCNE densities, HCNE locations, or both. Density plots for the different sets will be overlaid (Figure 2a), so that the plot for set two is drawn on top of that for set one, and the plot for set three drawn on top of that for set two. If the option to separate densities based on chromosomes in other genomes is used, the browser will attempt to create one density plot for each chromosome (in the other genome) for which there are HCNEs in the displayed region, or within half a sliding window to either side. If the resulting number of plots exceeds the number of plots requested on this configuration page, densities for the chromosomes with least HCNE sequence in this region will be combined into one plot labeled 'other' (Figure 5).

The Ancora genome browser was built using the GBrowse software [31], which is used by most model organism databases. The basic user interface should thus be familiar to most users. To visualize HCNE data in the most informative manner and to efficiently plot HCNE densities along entire chromosomes, we have extended GBrowse with a number of plugins and custom glyphs. The plugins that retrieve and render HCNE data can be configured by selecting a HCNE data set of interest from the 'Reports & Analysis' menu above the 'Scroll/Zoom' controls or by clicking on a HCNE density plot (Figure 2a). On the configuration page (Figure 3), the user can select which similarity thresholds to show HCNEs and HCNE densities for, and configure additional properties of the density plots (see below).

### Unique information revealed by HCNE density plots

**Figure 4:**
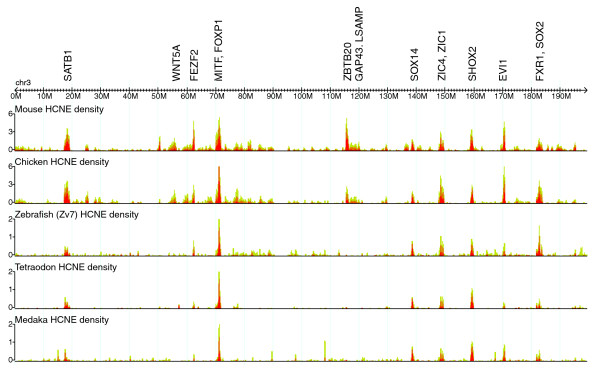
HCNE density distributions on human chromosome 3. Shown are densities of HCNEs identified from comparison with mouse, chicken and three different fish genomes. This genome browser screenshot has been manually labeled with likely target genes of HCNE enhancer activity at major density peaks. Target genes were identified by zooming in to inspect gene annotations at each peak.

Plots of HCNE density along chromosomes highlight regions that harbor large HCNE arrays and, thus, are likely to contain key developmental regulatory genes and correspond to regulatory domains [2,6,18,32,33]. Unlike conservation profiles, which can be seen in several other genome browsers [21,22,30,34], HCNE density plots do not directly reflect conservation on the sequence/alignment level; instead, they show density distributions of HCNEs on a larger scale. The result is qualitatively different from a sequence based conservation plot such as the Conservation track in the UCSC Genome Browser (Figure 2, compare (a) and (b)): it clearly reveals chromosomal regions of extensive noncoding conservation (Figure 4) and points to the approximate extent of GRBs, as well as the most likely target gene(s) within those regions [18].

We compute HCNE densities as the percentage of bases covered by HCNEs within a window of a given size. Because the genome browser computes HCNE densities on demand, the window size can be set by the user. The algorithm that computes the densities moves a window across the displayed chromosomal segment in steps of a size that is adapted to the size of the displayed segment. If the user zooms in to single-base resolution, densities are computed for every base shown. At lower resolutions, the step size is at least one step per pixel and ten steps per window. In our experience, this is more than sufficient for detecting peaks of interest. At resolutions where several density values are computed for each pixel, the plot shows the maximum density value per pixel, so that peaks are not omitted. By default, the browser displays overlaid density curves for HCNEs detected at three different sequence identity thresholds (Figure 2a). This allows users to easily locate regions with the most strongly conserved HCNEs and simultaneously delineate other HCNE-dense regions. The default window size for vertebrate genomes is 300 kb. It is important to note that this large window size leads to slopes of GRB signals extending outside the actual HCNE-spanned regions. To estimate the edges better, the user should consult synteny and HCNE location tracks, or decrease the window size in density plots. Despite this side effect, large window sizes are more appropriate for outlining GRB distribution along chromosomes, as well as for the determination of most likely target genes. It should also be noted that extremely high densities of HCNEs detected at the most stringent identity thresholds (high red density peaks) can originate from (rare) cross-species contamination of genome sequences. Users of the Ancora genome browser can identify such contamination as high HCNE densities coming from near-identical sequence segments confined to a single compared species. For example, much of *Xenopus tropicalis *scaffold 7291 is composed of fragments of near-identity to human chromosome 5, even though these regions have no HCNEs conserved in mouse, chicken or fish.

### Discovering genes that encode developmental regulators

Since there is a strong association between HCNE arrays and developmental regulatory genes [1-7], it is likely that most regions of high HCNE-density contain at least one developmental regulatory gene, even in cases where no such gene has been annotated. Inspection of HCNE density can thus be used to formulate hypotheses about gene function and identify likely target genes of putative enhancer activity of HCNEs. In a study from 2004, Sandelin *et al*. [2] identified HCNEs conserved among human, mouse and fugu, and closely inspected the 50 most HCNE-rich regions for the presence of developmental regulatory genes. They found 41 of these regions to contain a gene known to be involved in embryonic development. Of the remaining nine regions, seven contained a gene known to be a transcription factor or predicted as such based on homology. In a recent study, one of these transcription factor genes (FLJ20321) was recognized as a homolog of the *Drosophila *gene *castor *and found to be upregulated in cell differentiation [35], confirming the prediction from HCNE density. Sandelin *et al*. focused on the 50 HCNE-densest regions they detected in the human genome. Inspection of other HCNE-dense regions has revealed that several coincide with microRNA gene loci [18], a class of regulators implicated in multiple aspects of development [36]. We predict that many additional HCNE-dense regions will be found to contain developmental regulators. By plotting HCNE densities along entire chromosomes, the Ancora genome browser makes it easy to survey genomes for HCNE-dense regions (Figure 4). HCNE density curves from multiple pairwise genome comparisons can be shown simultaneously, so that users can identify regions rich in HCNEs that are specific to a subset of species, or shared across many species, if so desired. By zooming in, the user can investigate these regions in detail by inspecting the genome annotation available in Ancora as well as annotation in the other genome browsers to which direct links are provided. As a demonstration of the immediate utility of Ancora, we identified 129 genomic regions in the human genome in which the density of human-zebrafish HCNEs (70% identity over 50 columns) surpassed 0.5% and, using the principles outlined here, identified putative target genes in 120 of these regions (Additional data file 1). The regions in which no target gene could be assigned are prime candidates for discovery of novel genes or non-coding RNA involved in developmental regulation.

### Detecting and interpreting duplicated GRBs

**Figure 5:**
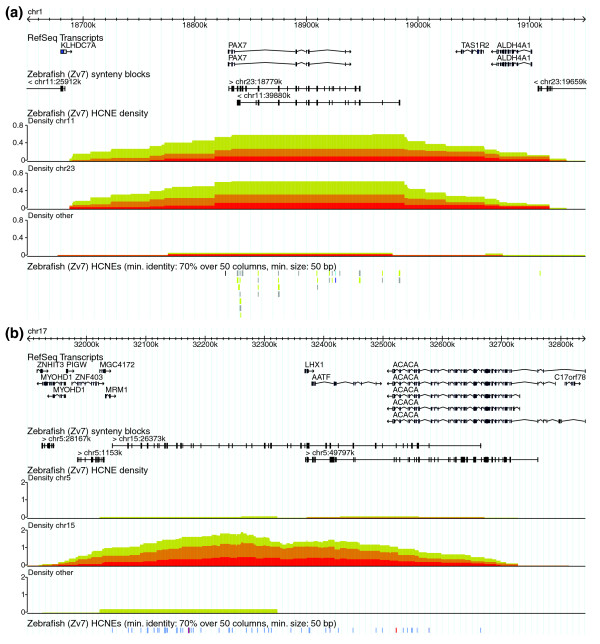
Duplicated GRBs. Zebrafish HCNEs and their density distribution are shown for the human **(a) ***PAX7 *and **(b) ***LHX1 *loci. HCNEs are colored by the zebrafish chromosome they map to. In (a), most HCNEs are colored light green or gray and map to zebrafish chromosomes 11 or 23, respectively. In (b), most HCNEs are colored blue and map to zebrafish chromosome 15 (because this region contains many HCNEs, they are collapsed on a single row in this screenshot). The density plots are also separated based on zebrafish chromosomes. Comparison of synteny blocks to exon locations indicate that orthologs of *AATF *and *ACACA *are present next to the *LHX1 *ortholog (*lhx1b*) on zebrafish chromosome 5, but not on zebrafish chromosome 15 where *lhx1a *is located; this can be confirmed by detailed inspection of the zebrafish loci.

As a result of whole-genome duplication in teleosts, many mammalian GRBs have two orthologous GRBs in teleost genomes. The Ancora genome browser makes it easy to locate such GRBs by coloring HCNEs according to the chromosome they align to in the other genome. For example, when viewing human-zebrafish HCNEs along human chromosomes, the hallmark of a GRB present in two copies in zebrafish is a HCNE-dense region where HCNEs occur mainly in two different colors. Such regions can also be discovered by activating an option that makes the genome browser separate HCNE density plots based on chromosomes in the other genome (Figure 3). Figure 5a shows an example: the GRB of *PAX7*, a transcription factor gene implicated in muscle development [37] and situated within an array of HCNEs. Most of the human-zebrafish HCNEs in this region are colored either gray or light green in the genome browser (Figure 5a) and align to orthologous loci on zebrafish chromosomes 23 and 11, respectively. Thus, this view quickly suggests that noncoding putative regulatory sequences have been preserved to a similar extent at both of the *pax7 *loci in zebrafish. In contrast, Figure 5b shows an example where duplicate GRBs have diverged to a large extent in zebrafish. Human *LHX1*, a LIM homeobox transcription factor gene implicated in head, neural and reproductive development [38] is within an array of HCNEs that extends into the neighboring genes *AATF*, which encodes a transcription factor involved in cell cycle control, and *ACACA*, which encodes a carboxylase involved in fatty acid synthesis. Most of the human-zebrafish HCNEs in this region are colored blue in the genome browser (Figure 5b) and align to the region around *lhx1a *on zebrafish chromosome 15. Thus, noncoding putative regulatory sequences appear to have been preserved to a much larger extent around *lhx1a *than around *1hx1b*. A detailed inspection of the zebrafish loci reveals that orthologs of *AATF *and *ACACA *have been retained at the *lhx1b *locus, but lost from the *lhx1a *locus, where there are more HCNEs (in Ancora, the zebrafish loci can be inspected by jumping from the displayed human locus to corresponding loci in the zebrafish genome by clicking on HCNEs or synteny blocks). Following the rationale in [18] this confirms that the HCNE array is unrelated to *AATF *and *ACACA*, and allows the classification of these two genes as bystanders.

### Distinguishing chromosomal regulatory domains

**Figure 6:**
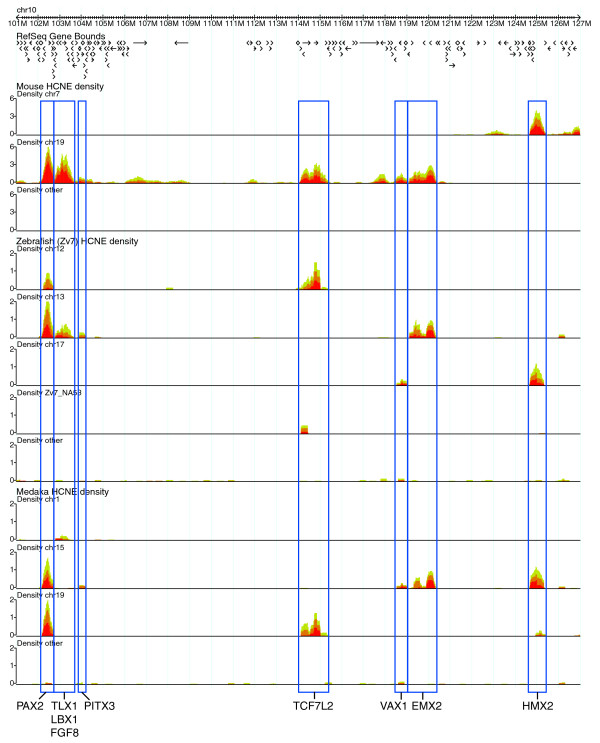
Distinguishing regulatory domains. Screenshot from the Ancora genome browser showing 26 Mb of human chromosome 10 and HCNE densities from comparisons with mouse, zebrafish and medaka. HCNE density curves are separated based on chromosomes in these organisms (Zv7_NA53 is a contig that has not been assigned to a chromosome). To illustrate the use of this view for distinguishing chromosomal regulatory domains, rectangles have been manually added to the screenshot around density peaks indicating clusters of HCNEs in conserved synteny. Rectangles are labeled with regulatory genes annotated in the corresponding genomic regions.

By comparing HCNE arrays and synteny blocks, we have observed that the extent of a HCNE array often provides a good approximation of the extent of the corresponding GRB [6,18]. However, unless synteny conservation is taken into account, partitioning of HCNEs into separate arrays becomes arbitrary in regions with high noncoding conservation. In the Ancora genome browser, it is easy to visualize synteny conservation of HCNE arrays over large genomic segments by activating the option that separates HCNE density plots based on chromosome in the other genome. The result is an overview of how HCNE-dense regions have been partitioned over different chromosomes in evolution (Figure 6). Based on the assumption that fundamental regulatory domains have been maintained in evolution [6,15-18], the displayed separation of HCNE-dense regions across chromosomes should correspond to a separation of distinct regulatory domains. We expect the resolution of this approach to increase as more genomes are sequenced and assembled.

## Viewing HCNEs and density plots in other genome browsers

**Figure 7:**
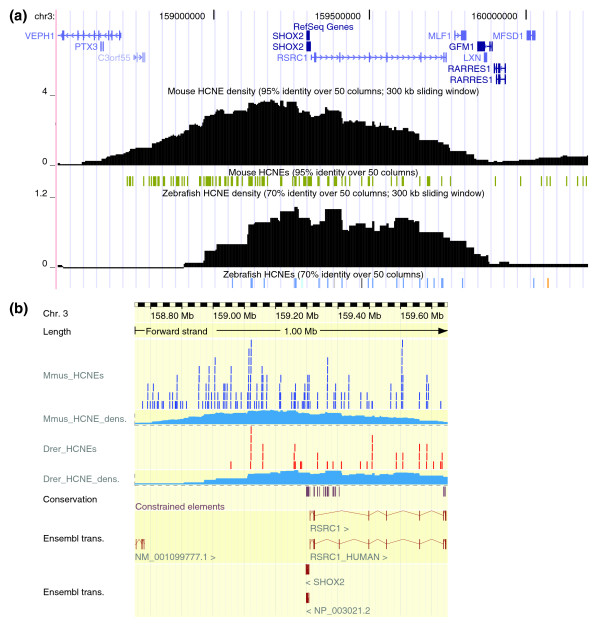
Ancora tracks in the UCSC and Ensembl genome browsers. Genome browser views of region around the human *SHOX2 *gene. Added tracks show locations and densities for HCNEs detected at similarity thresholds of 95% in the mouse comparison and 70% in the zebrafish comparison. **(a) **UCSC, same region as in Figure 2. **(b) **Ensembl, displaying 1 Mb (the maximum allowed size in ContigView) of the same region. Additional tracks from Ensembl show conserved elements ('Conservation') and transcripts ('Ensembl trans.').

The Ancora genome browser provides the most flexible way to explore the HCNE data in Ancora. However, it is often useful to view these data in other browsers where it can be compared to other types of annotation. We aimed to make it as straightforward as possible to view HCNE data in the UCSC Genome Browser [21] and Ensembl [22] (Figure 7).

HCNE locations and precomputed density curves are available for download in the 'bed' and 'wig' formats used for UCSC Genome Browser custom tracks [39]. It is not necessary to download the .bed and .wig files to use them as custom tracks in the UCSC Genome Browser: the user can simply copy the URLs for track files of interest from the Ancora downloads section and paste them into the 'add custom tracks' form on the UCSC Genome Browser web site.

The Ensembl browser can display sequence annotations provided over the web through DAS, a method for data exchange [23]. Much of the Ancora data are available through DAS. Ancora provides an interface where the user can add HCNE tracks to Ensembl ContigView. Tracks added in this way are stored as part of the user's Ensembl preferences. Users who are familiar with DAS can also retrieve data directly from the DAS server. For example, the URL given in reference [40] provides a list of available tracks.

## Comparison to other tools

While the genome browsers at UCSC and Ensembl provide rich and diverse annotation sets including information about sequence conservation, they do not distinguish coding from noncoding conserved elements. To our knowledge, the Ancora genome browser is the first tool that makes it easy to visualize HCNE distributions on large genomic regions, up to whole chromosomes, and the browser is tailored to show data in a flexible manner at this level.

The ECR Browser [34] and VISTA Browser [30] allow detailed inspection of sequence conservation profiles across many genomes, highlight conserved elements in a user-customizable manner and distinguish noncoding from coding conservation. In the ECR Browser, one drawback is that thresholds for detection of conserved elements are uniform across all comparisons shown, irrespective of evolutionary distance. In contrast, Ancora and VISTA browsers can show results for multiple different thresholds simultaneously. A limitation of both the ECR and VISTA browsers is that they are not designed for visualizing the distribution of conserved elements on segments larger than a few megabases. The VISTA Browser can only display regions up to 5 Mb in size and the ECR Browser's display of large regions is difficult to interpret because conserved elements are drawn close together. In contrast, the HCNE density plots in Ancora make it possible to view and intuitively interpret HCNE content at any scale. Ancora is therefore better suited for exploring conservation genome-wide and discovering regulatory domains at loci not known beforehand, while the ECR and VISTA browsers provide more functionality for close examination of sequence-level conservation profiles.

The CONDOR database [14] holds information on about 6,800 HCNEs from about 120 blocks of conserved synteny between human and fugu and provides a graphical interface to view the distribution of HCNEs in those regions. While there are several similarities between Ancora and CONDOR, Ancora has the advantage of providing HCNE data for entire genomes. Another difference between the two resources is that the Ancora HCNE sets are not as stringently defined in terms of conservation as those in CONDOR, where HCNEs are required to be conserved among four diverged vertebrates. In Ancora, we have chosen to provide a range of HCNE data sets from different pairwise comparisons and with different similarity thresholds (Figure 1 and Table 1), so that users can choose to look at the data appropriate for their questions. A valuable section of CONDOR provides developmental expression patterns for about 100 HCNEs that have been investigated by reporter assays in zebrafish. We are preparing to link similar data to Ancora.

## Summary

Ancora is a new web resource that provides data and tools for exploring HCNEs and their association with developmental regulatory genes. Built upon a database of HCNEs conserved between various metazoan genomes, Ancora provides a genome browser for visualizing the distribution of those elements on chromosomes in the context of other types of annotation integrated from different sources. One of the novel features of Ancora is the possibility to display highly customizable plots of HCNE density along chromosomes. HCNE density plots are qualitatively different from conservation profiles available in other genome browsers [21,22,30,34]: they clearly reveal regions of extensive noncoding conservation and highlight larger chromosomal regulatory domains (GRBs) that have been maintained in evolution. The GRBs typically coincide with loci of developmental regulatory genes, for which HCNEs appear to act as enhancers [3,8-12]. Consequently, we anticipate that Ancora will be highly useful for discovering developmental regulatory genes and their distal *cis*-regulatory elements. We have illustrated how Ancora can be used to define the chromosomal regulatory domains of those genes and distinguish genes that appear to be functionally associated with HCNEs from unrelated 'bystander' genes within the same GRB. The HCNE data in Ancora are also available for download and can easily be displayed in the popular general-purpose genome browsers at UCSC [21] and Ensembl [22].

## Abbreviations

DAS, distributed annotation system; GRB, genomic regulatory block; HCNE, highly conserved noncoding element.

## Authors' contributions

BL conceived of and supervised the study. PGE and DF designed and implemented the pipeline for detecting HCNEs. PGE implemented the web resource. All authors participated in writing the paper and approved the final version.

## Additional data files

The following additional data are available with the online version of this paper. Human genomic regions in which the density (in a 300 kb sliding window) of human-zebrafish HCNEs (70% identity over 50 columns) surpassed 0.5% and putative target genes in 120 of these regions.

## Supplementary Material

Additional data file 1 Human genomic regions in which the density (in a 300 kb sliding window) of human-zebrafish HCNEs (70% identity over 50 columns) surpassed 0.5% and putative target genes in 120 of these regions. Click here for file

## References

[B1] Bejerano G, Pheasant M, Makunin I, Stephen S, Kent WJ, Mattick JS, Haussler D (2004). Ultraconserved elements in the human genome.. Science.

[B2] Sandelin A, Bailey P, Bruce S, Engström PG, Klos JM, Wasserman WW, Ericson J, Lenhard B (2004). Arrays of ultraconserved non-coding regions span the loci of key developmental genes in vertebrate genomes.. BMC Genomics.

[B3] Woolfe A, Goodson M, Goode DK, Snell P, McEwen GK, Vavouri T, Smith SF, North P, Callaway H, Kelly K, Walter K, Abnizova I, Gilks W, Edwards YJ, Cooke JE, Elgar G (2005). Highly conserved non-coding sequences are associated with vertebrate development.. PLoS Biol.

[B4] Venkatesh B, Kirkness EF, Loh YH, Halpern AL, Lee AP, Johnson J, Dandona N, Viswanathan LD, Tay A, Venter JC, Strausberg RL, Brenner S (2006). Ancient noncoding elements conserved in the human genome.. Science.

[B5] Glazov EA, Pheasant M, McGraw EA, Bejerano G, Mattick JS (2005). Ultraconserved elements in insect genomes: a highly conserved intronic sequence implicated in the control of homothorax mRNA splicing.. Genome Res.

[B6] Engström PG, Ho Sui SJ, Drivenes Ø, Becker TS, Lenhard B (2007). Genomic regulatory blocks underlie extensive microsynteny conservation in insects.. Genome Res.

[B7] Vavouri T, Walter K, Gilks WR, Lehner B, Elgar G (2007). Parallel evolution of conserved non-coding elements that target a common set of developmental regulatory genes from worms to humans.. Genome Biol.

[B8] Nobrega MA, Ovcharenko I, Afzal V, Rubin EM (2003). Scanning human gene deserts for long-range enhancers.. Science.

[B9] Kimura-Yoshida C, Kitajima K, Oda-Ishii I, Tian E, Suzuki M, Yamamoto M, Suzuki T, Kobayashi M, Aizawa S, Matsuo I (2004). Characterization of the pufferfish Otx2 cis-regulators reveals evolutionarily conserved genetic mechanisms for vertebrate head specification.. Development.

[B10] de la Calle-Mustienes E, Feijoo CG, Manzanares M, Tena JJ, Rodriguez-Seguel E, Letizia A, Allende ML, Gomez-Skarmeta JL (2005). A functional survey of the enhancer activity of conserved non-coding sequences from vertebrate Iroquois cluster gene deserts.. Genome Res.

[B11] Pennacchio LA, Ahituv N, Moses AM, Prabhakar S, Nobrega MA, Shoukry M, Minovitsky S, Dubchak I, Holt A, Lewis KD, Plajzer-Frick I, Akiyama J, De Val S, Afzal V, Black BL, Couronne O, Eisen MB, Visel A, Rubin EM (2006). *In vivo *enhancer analysis of human conserved non-coding sequences.. Nature.

[B12] Shin JT, Priest JR, Ovcharenko I, Ronco A, Moore RK, Burns CG, MacRae CA (2005). Human-zebrafish non-coding conserved elements act *in vivo *to regulate transcription.. Nucleic Acids Res.

[B13] Visel A, Minovitsky S, Dubchak I, Pennacchio LA (2007). VISTA Enhancer Browser - a database of tissue-specific human enhancers.. Nucleic Acids Res.

[B14] Woolfe A, Goode DK, Cooke J, Callaway H, Smith S, Snell P, McEwen GK, Elgar G (2007). CONDOR: a database resource of developmentally associated conserved non-coding elements.. BMC Dev Biol.

[B15] Ahituv N, Prabhakar S, Poulin F, Rubin EM, Couronne O (2005). Mapping cis-regulatory domains in the human genome using multi-species conservation of synteny.. Hum Mol Genet.

[B16] Kleinjan DA, van Heyningen V (2005). Long-range control of gene expression: emerging mechanisms and disruption in disease.. Am J Hum Genet.

[B17] Becker TS, Lenhard B (2007). The random versus fragile breakage models of chromosome evolution: a matter of resolution.. Mol Genet Genomics.

[B18] Kikuta H, Laplante M, Navratilova P, Komisarczuk AZ, Engström PG, Fredman D, Akalin A, Caccamo M, Sealy I, Howe K, Ghislain J, Pezeron G, Mourrain P, Ellingsen S, Oates AC, Thisse C, Thisse B, Foucher I, Adolf B, Geling A, Lenhard B, Becker TS (2007). Genomic regulatory blocks encompass multiple neighboring genes and maintain conserved synteny in vertebrates.. Genome Res.

[B19] Jaillon O, Aury JM, Brunet F, Petit JL, Stange-Thomann N, Mauceli E, Bouneau L, Fischer C, Ozouf-Costaz C, Bernot A, Nicaud S, Jaffe D, Fisher S, Lutfalla G, Dossat C, Segurens B, Dasilva C, Salanoubat M, Levy M, Boudet N, Castellano S, Anthouard V, Jubin C, Castelli V, Katinka M, Vacherie B, Biémont C, Skalli Z, Cattolico L, Poulain J (2004). Genome duplication in the teleost fish *Tetraodon nigroviridis *reveals the early vertebrate proto-karyotype.. Nature.

[B20] Ancora. http://ancora.genereg.net.

[B21] Kuhn RM, Karolchik D, Zweig AS, Trumbower H, Thomas DJ, Thakkapallayil A, Sugnet CW, Stanke M, Smith KE, Siepel A, Rosenbloom KR, Rhead B, Raney BJ, Pohl A, Pedersen JS, Hsu F, Hinrichs AS, Harte RA, Diekhans M, Clawson H, Bejerano G, Barber GP, Baertsch R, Haussler D, Kent WJ (2007). The UCSC genome browser database: update 2007.. Nucleic Acids Res.

[B22] Hubbard TJ, Aken BL, Beal K, Ballester B, Caccamo M, Chen Y, Clarke L, Coates G, Cunningham F, Cutts T, Down T, Dyer SC, Fitzgerald S, Fernandez-Banet J, Graf S, Haider S, Hammond M, Herrero J, Holland R, Howe K, Howe K, Johnson N, Kahari A, Keefe D, Kokocinski F, Kulesha E, Lawson D, Longden I, Melsopp C, Megy K (2007). Ensembl 2007.. Nucleic Acids Res.

[B23] Dowell RD, Jokerst RM, Day A, Eddy SR, Stein L (2001). The distributed annotation system.. BMC Bioinformatics.

[B24] Kent WJ, Baertsch R, Hinrichs A, Miller W, Haussler D (2003). Evolution's cauldron: duplication, deletion, and rearrangement in the mouse and human genomes.. Proc Natl Acad Sci USA.

[B25] Wheeler DL, Barrett T, Benson DA, Bryant SH, Canese K, Chetvernin V, Church DM, DiCuccio M, Edgar R, Federhen S, Geer LY, Kapustin Y, Khovayko O, Landsman D, Lipman DJ, Madden TL, Maglott DR, Ostell J, Miller V, Pruitt KD, Schuler GD, Sequeira E, Sherry ST, Sirotkin K, Souvorov A, Starchenko G, Tatusov RL, Tatusova TA, Wagner L, Yaschenko E (2007). Database resources of the National Center for Biotechnology Information.. Nucleic Acids Res.

[B26] Eppig JT, Blake JA, Bult CJ, Kadin JA, Richardson JE (2007). The mouse genome database (MGD): new features facilitating a model system.. Nucleic Acids Res.

[B27] Sprague J, Bayraktaroglu L, Bradford Y, Conlin T, Dunn N, Fashena D, Frazer K, Haendel M, Howe DG, Knight J, Mani P, Moxon SA, Pich C, Ramachandran S, Schaper K, Segerdell E, Shao X, Singer A, Song P, Sprunger B, Van Slyke CE, Westerfield M (2007). The Zebrafish Information Network: the zebrafish model organism database provides expanded support for genotypes and phenotypes.. Nucleic Acids Res.

[B28] Crosby MA, Goodman JL, Strelets VB, Zhang P, Gelbart WM (2007). FlyBase: genomes by the dozen.. Nucleic Acids Res.

[B29] Griffiths-Jones S, Grocock RJ, van Dongen S, Bateman A, Enright AJ (2006). miRBase: microRNA sequences, targets and gene nomenclature.. Nucleic Acids Res.

[B30] Brudno M, Poliakov A, Minovitsky S, Ratnere I, Dubchak I (2007). Multiple whole genome alignments and novel biomedical applications at the VISTA portal.. Nucleic Acids Res.

[B31] Stein LD, Mungall C, Shu S, Caudy M, Mangone M, Day A, Nickerson E, Stajich JE, Harris TW, Arva A, Lewis S (2002). The generic genome browser: a building block for a model organism system database.. Genome Res.

[B32] Lindblad-Toh K, Wade CM, Mikkelsen TS, Karlsson EK, Jaffe DB, Kamal M, Clamp M, Chang JL, Kulbokas EJ, Zody MC, Mauceli E, Xie X, Breen M, Wayne RK, Ostrander EA, Ponting CP, Galibert F, Smith DR, DeJong PJ, Kirkness E, Alvarez P, Biagi T, Brockman W, Butler J, Chin CW, Cook A, Cuff J, Daly MJ, DeCaprio D, Gnerre S (2005). Genome sequence, comparative analysis and haplotype structure of the domestic dog.. Nature.

[B33] Mikkelsen TS, Wakefield MJ, Aken B, Amemiya CT, Chang JL, Duke S, Garber M, Gentles AJ, Goodstadt L, Heger A, Jurka J, Kamal M, Mauceli E, Searle SM, Sharpe T, Baker ML, Batzer MA, Benos PV, Belov K, Clamp M, Cook A, Cuff J, Das R, Davidow L, Deakin JE, Fazzari MJ, Glass JL, Grabherr M, Greally JM, Gu W (2007). Genome of the marsupial *Monodelphis domestica *reveals innovation in non-coding sequences.. Nature.

[B34] Ovcharenko I, Nobrega MA, Loots GG, Stubbs L (2004). ECR Browser: a tool for visualizing and accessing data from comparisons of multiple vertebrate genomes.. Nucleic Acids Res.

[B35] Liu Z, Yang X, Tan F, Cullion K, Thiele CJ (2006). Molecular cloning and characterization of human Castor, a novel human gene upregulated during cell differentiation.. Biochem Biophys Res Commun.

[B36] Zhao Y, Srivastava D (2007). A developmental view of microRNA function.. Trends Biochem Sci.

[B37] Buckingham M (2007). Skeletal muscle progenitor cells and the role of Pax genes.. C R Biol.

[B38] Hunter CS, Rhodes SJ (2005). LIM-homeodomain genes in mammalian development and human disease.. Mol Biol Rep.

[B39] UCSC Genome Bioinformatics - FAQ: Data File Formats. http://genome.ucsc.edu/FAQ/FAQformat.

[B40] Ancora DAS Tracks. http://ancora.genereg.net/das/dsn.

[B41] International Chicken Genome Sequencing Consortium (2004). Sequence and comparative analysis of the chicken genome provide unique perspectives on vertebrate evolution.. Nature.

[B42] Aparicio S, Chapman J, Stupka E, Putnam N, Chia JM, Dehal P, Christoffels A, Rash S, Hoon S, Smit A, Gelpke MD, Roach J, Oh T, Ho IY, Wong M, Detter C, Verhoef F, Predki P, Tay A, Lucas S, Richardson P, Smith SF, Clark MS, Edwards YJ, Doggett N, Zharkikh A, Tavtigian SV, Pruss D, Barnstead M, Evans C (2002). Whole-genome shotgun assembly and analysis of the genome of *Fugu rubripes*.. Science.

[B43] Kasahara M, Naruse K, Sasaki S, Nakatani Y, Qu W, Ahsan B, Yamada T, Nagayasu Y, Doi K, Kasai Y, Jindo T, Kobayashi D, Shimada A, Toyoda A, Kuroki Y, Fujiyama A, Sasaki T, Shimizu A, Asakawa S, Shimizu N, Hashimoto S, Yang J, Lee Y, Matsushima K, Sugano S, Sakaizumi M, Narita T, Ohishi K, Haga S, Ohta F (2007). The medaka draft genome and insights into vertebrate genome evolution.. Nature.

[B44] Celniker SE, Wheeler DA, Kronmiller B, Carlson JW, Halpern A, Patel S, Adams M, Champe M, Dugan SP, Frise E, Hodgson A, George RA, Hoskins RA, Laverty T, Muzny DM, Nelson CR, Pacleb JM, Park S, Pfeiffer BD, Richards S, Sodergren EJ, Svirskas R, Tabor PE, Wan K, Stapleton M, Sutton GG, Venter C, Weinstock G, Scherer SE (2002). Finishing a whole-genome shotgun: release 3 of the *Drosophila melanogaster *euchromatic genome sequence.. Genome Biol.

[B45] Richards S, Liu Y, Bettencourt BR, Hradecky P, Letovsky S, Nielsen R, Thornton K, Hubisz MJ, Chen R, Meisel RP, Couronne O, Hua S, Smith MA, Zhang P, Liu J, Bussemaker HJ, van Batenburg MF, Howells SL, Scherer SE, Sodergren E, Matthews BB, Crosby MA, Schroeder AJ, Ortiz-Barrientos D, Rives CM, Metzker ML, Muzny DM, Scott G, Steffen D, Wheeler DA (2005). Comparative genome sequencing of *Drosophila pseudoobscura*: chromosomal, gene, and cis-element evolution.. Genome Res.

[B46] Clark AG, Eisen MB, Smith DR, Bergman CM, Oliver B, Markow TA, Kaufman TC, Kellis M, Gelbart W, Iyer VN, Pollard DA, Sackton TB, Larracuente AM, Singh ND, Abad JP, Abt DN, Adryan B, Aguade M, Akashi H, Anderson WW, Aquadro CF, Ardell DH, Arguello R, Artieri CG, Barbash DA, Barker D, Barsanti P, Batterham P, Batzoglou S, Drosophila 12 Genomes Consortium (2007). Evolution of genes and genomes on the *Drosophila *phylogeny.. Nature.

[B47] Cobb J, Dierich A, Huss-Garcia Y, Duboule D (2006). A mouse model for human short-stature syndromes identifies Shox2 as an upstream regulator of Runx2 during long-bone development.. Proc Natl Acad Sci USA.

